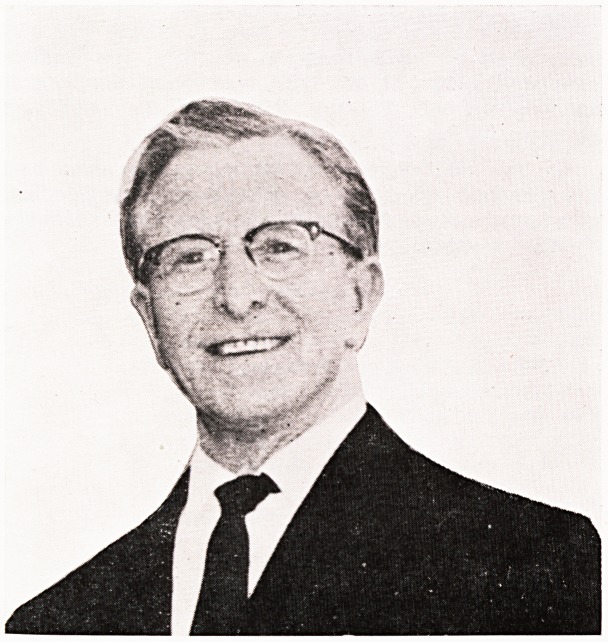# A. E. S. Roberts

**Published:** 1971-10

**Authors:** 


### Obituary

### A. E. S. ROBERTS, M.A.

The sudden death of Mr. A. E. S. Roberts, M.A., on Monday, 5th April 1971, came as a severe shock to his many friends in the University of Bristol and outside in the wider circle of the Bristol Medico-Chirurgical Society. The previous Friday he had been paying one of his regular visits to old friends in the University and had seemed in the best of health, obviously enjoying every minute of his retirement near Minehead and brimming over with plans for the summer months ahead.

Austin Edward Spencer Roberts joined the University Library staff as an assistant in the Medical Library on the 19th March 1925, a few months before the building now known as the Wills Memorial Building was formally opened by King George V in company with his consort. The then University Librarian was concerned to have an all-male library staff and Roberts was the first of a line of juniors recruited for a time exclusively from Queen Elizabeth Hospital. On the death of the Assistant Librarian in charge of the Medical Library in 1930, Roberts was placed in charge of the Medical Library. In addition to duties as Medical Librarian he acted as Secretary to the Library and recorded and processed all books and periodicals, medical and otherwise, added to the University Library as well as attending to the library finances. In 1941 he joined the Army and served with distinction with the 8th Army in North Africa. Upon his return he was offered the choice of employment either as Medical Librarian or as Library Secretary and unhesitatingly chose the former to the advantage of both Library and Medical School. He reclassified the Library and was a prominent member of the then newly-formed Medical section of the Library Association. After the war the Medical Library developed steadily and rapidly under his wise guidance. The setting up of the Veterinary School in 1947 meant a great deal of work for him in building up a veterinary library from scratch and in 1950 the holdings of the Medical Library were impressively enriched by an outstanding collection of early medical books which came from the Royal United Hospital, Bath and which had been gathered together by Bath medical men, notably Caleb Hillier Parry and John Smith Soden. Roberts tended these with loving care and set about organizing the repair and restoration of these books, aided by a generous subvention from the University funds. Much later he was instrumental in persuading the Wellcome Trust to offer a further £1,800 to complete the rebinding of these volumes.

In 1963, Stage I of a new Medical School was completed and, after about 40 years in the Wills Memorial Building the Medical Library moved to splendid new quarters in the new building. Roberts was concerned at every stage with the detailed planning of the new Medical Library and supervised the move. So wholeheartedly did he enter into the moving operations that he had to be ordered home for a rest at one point as he was in imminent danger of incapacitating himself through exhaustion. His services to the Medical Library were fittingly recognized by the University when it awarded him the degree of M.A., honoris causa in 1957. By special permission he was allowed to retire on his 65th birthday.

Although he possessed no formal library qualifications nor had undergone training at Library School, Roberts was a natural librarian. He was outstandingly efficient and conscientious to a degree. He knew the Medical library stock like the back of his hand and could answer many questions off the cuff without recourse to the catalogue. The present writer never ceased to marvel at the detailed accuracy of his bibliographical knowledge. Titles, editions, dates, authors and editors he could reel off with deceptive ease. He had an amazing gift for inspiring in the reader, after a remarkably short space of time, a degree of confidence in his librarian's judgment, knowledge and authority. To this was allied a sympathetic, understanding approach to each individual reader's particular problems. To some consultants of the old school, no enquiry, however simple or trivial, was satisfactorily answered unless Mr. Roberts had been called to the telephone to deal with it personally. Always helpful, he greatly assisted hospital librarians in the South West and the Medical Library was the focal point in the area for medical literature and information. He was one of the best known and best liked members of the Medical section of the Library Association. In 1963 he attended the first International Congress on Medical Librarianship held in Washington, U.S.A.

Painstaking to a degree, nothing was too much trouble for him where his beloved Medical Library was concerned. He would stay with each new junior assistant on his/her first evening duty in the Library to make certain that all was well and during his 45 years service had the satisfaction of seeing several of his "boys" (i.e. assistants) rise to positions of eminence in the Academic Library world; a tribute to his great patience and skill in grounding them in the fundamentals of the profession. His zest for living, particularly in his native Bristol, his enthusiasm for seeing the best in the best of all possible worlds and his sense of humour are not forgotten. His delight and glee at the chance discovery, late in his career, that the Index Medicus, which somehow kept arriving at the University Medical Library year after year without request for payment, was in fact sent in exchange for the Bristol Medico-Chirurgical Journal (when such exchanges are normally conducted on a strictly equivalent *quid pro quo*), remains fresh in the memory.

The 'Med.-Chi.' Society, and particularly its Honorary Secretaries, had cause to be eternally grateful to him for his constant efficiency and conscientiousness in keeping alive the accurate index of their members, and for sending out the monthly notices of meetings. He knew the history and organisation of the Society intimately and any appeal for help or advice was met immediately and with unfailing courtesy. The Society were well aware of the debt they owed him and it was with great pleasure that they met half the expenses for his trip to America in 1963.

His trim neat figure with twinkling blue eyes and firm jaunty step is sorely missed but the Medical Library remains—a monument and tribute to his labours of forty-five years.

## Figures and Tables

**Figure f1:**